# Small molecule ice recrystallization inhibitors mitigate red blood cell lysis during freezing, transient warming and thawing

**DOI:** 10.1038/srep23619

**Published:** 2016-03-29

**Authors:** Jennie G. Briard, Jessica S. Poisson, Tracey R. Turner, Chantelle J. Capicciotti, Jason P. Acker, Robert N. Ben

**Affiliations:** 1 Department of Chemistry, University of Ottawa, Ottawa, ON, K1N 6N5, Canada; 2 Canadian Blood Services, Centre of Innovation, 8249–114 Street NW, Edmonton, AB, T6G 2R8, Canada

## Abstract

During cryopreservation, ice recrystallization is a major cause of cellular damage. Conventional cryoprotectants such as dimethyl sulfoxide (DMSO) and glycerol function by a number of different mechanisms but do not mitigate or control ice recrystallization at concentrations utilized in cryopreservation procedures. In North America, cryopreservation of human red blood cells (RBCs) utilizes high concentrations of glycerol. RBC units frozen under these conditions must be subjected to a time-consuming deglycerolization process after thawing in order to remove the glycerol to <1% prior to transfusion thus limiting the use of frozen RBC units in emergency situations. We have identified several low molecular mass ice recrystallization inhibitors (IRIs) that are effective cryoprotectants for human RBCs, resulting in 70–80% intact RBCs using only 15% glycerol and slow freezing rates. These compounds are capable of reducing the average ice crystal size of extracellular ice relative to a 15% glycerol control validating the positive correlation between a reduction in ice crystal size and increased post-thaw recovery of RBCs. The most potent IRI from this study is also capable of protecting frozen RBCs against the large temperature fluctuations associated with transient warming.

Cryopreservation remains the most common method for the long-term storage of various cells. However, during the cryopreservation process a significant number of cells experience irreparable damage^1^ due to the growth of ice ultimately resulting in decreased post-thaw recoveries or impaired function^1^,^2^,^3^,^4^,^5^. For instance, it has been demonstrated that at least 20% of all patients receiving hematopoietic stem cell transplants will experience primary graft failure as a direct result of reduced post-thaw viability and functionality of the CD34+ cells^6^. Thus, as cell-based therapeutics continue to define new models of care in stem cell therapy, regenerative medicine and transfusion, it is becoming increasingly important to ensure the highest level of post-thaw viability and functionality.

The most significant short fall of current cryopreservation protocols making them suboptimal is the failure to control ice recrystallization. *Ice recrystallization is the process that occurs during freezing and thawing whereby large ice crystals increase in size at the expense of smaller ones*. This process occurs primarily during warming/thawing of a frozen sample and the subsequent cellular damage it causes is the primary source of cell injury during cryopreservation^7^.

RBC transfusions are lifesaving for patients suffering from leukemias, hemolytic anemias, and from traumas resulting in severe blood loss. Cryopreservation is the only technology allowing access to large quantities of RBC units necessary when high numbers of RBC transfusions are required. However, the cryopreservation of RBCs for routine transfusion is not a common practice because current protocols do not permit direct transfusion immediately after thawing. Clinical cryopreservation protocols utilize high concentrations of glycerol (40% v/v) as a cryoprotectant however, after thawing time-consuming deglycerolization procedures are necessary to prevent intravascular hemolysis.

Early work in our laboratory identified a class of carbon-linked (*C*-linked) antifreeze glycoprotein (AFGP) analogues (Fig. 1) that possess custom-tailored antifreeze activity^8^,^9^. While these compounds are very effective inhibitors of ice recrystallization and excellent cryoprotectants for human liver cell lines in the absence of DMSO, they are not amenable to the large-scale synthesis necessary to prepare sufficient quantities for cryopreservation applications^10^. More recently, extensive structure-function studies performed in our laboratory revealed several low molecular weight (<340 Daltons) carbohydrate derivatives were effective inhibitors of ice recrystallization^11^,^12^,^13^,^14^,^15^. Several of these small molecules are effective additives for the freezing of human RBCs resulting in significantly higher numbers of intact RBCs post-thaw while using greatly reduced quantities of glycerol with slow freezing rates^16^. These small molecule inhibitors of ice recrystallization constitute a novel class of cryoprotective agents that may meet the increasing needs for long-term storage of important biological materials for emerging cell therapeutics in the field of regenerative medicine and tissue engineering.

In this paper, we demonstrate that these IRIs are capable of mitigating ice growth *in vitro* with reduced quantities of glycerol and this ability is correlated to increased post-thaw viability using annucleate human RBCs as an appropriate model. We also demonstrate that these compounds can protect cells against injury resulting from ice recrystallization during transient warming events (TWEs). TWEs have been recognized as another significant contributor to reduced post-thaw viabilities in sperm^17^, placental cord blood^18^,^19^,^20^, peripheral blood mononuclear cells^21^,^22^ and tissue allographs^23^. These attributes make small molecule inhibitors of ice recrystallization very valuable as novel cryoprotectants with a unique mode of action.

## Results

### *In Vitro* Effects of Small Molecule IRIs

**Figure 2:**
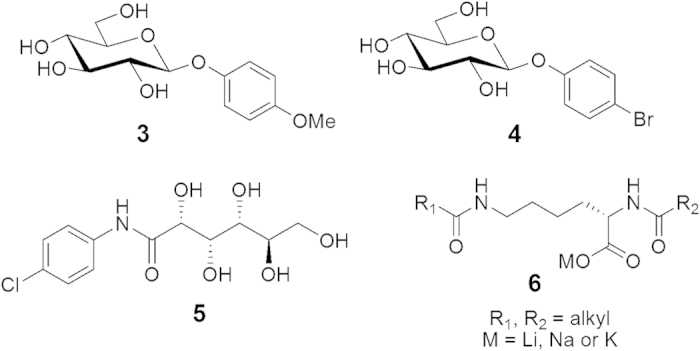
Chemical structure of aryl-glycosides (3,4), aryl-aldonamide (5) and a lysine-based non-ionic surfactant (6).

We have identified several different classes of small molecules that inhibit ice recrystallization (Fig. 2). These include aryl-glycosides (**3**,**4**), aryl-aldonamide **5** and lysine-based non-ionic surfactants with the general structure of **6**^16^,^24^.

**Figure 3:**
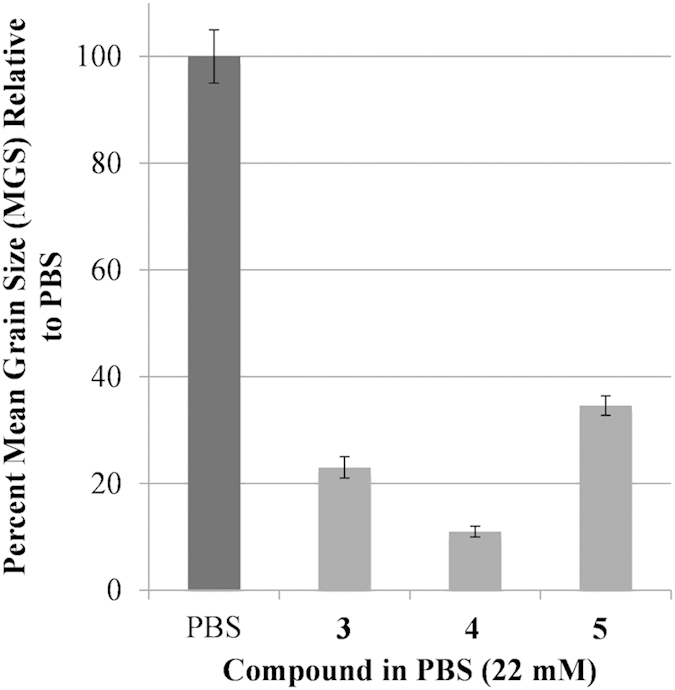
IRI activity of small molecules 3–5 at 22 mM. IRI activity is represented as a percent mean grain size (% MGS) after 30 minutes of recrystallization at −6.4 °C compared to a phosphate buffered saline (PBS) positive control for ice recrystallization.

The ability of **3** and **4** to inhibit ice recrystallization has been previously quantified and the IRI activity of **5** is shown in Fig. 3. Aldonamide **5** is the least active of the three compounds and substitution of the *para*-methoxy substituent in **3** with a bromine atom results in a potent inhibitor of ice recrystallization^4^.

**Figure 4:**
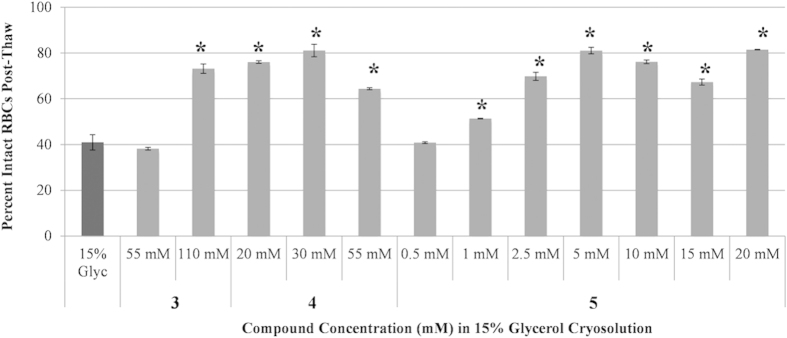
Optimization of IRI (3–5) concentration for freezing of human RBCs using 15% glycerol. RBCs were incubated for 10 minutes with 15% glycerol or 15% glycerol with compound **3**, **4**, or **5** at various concentrations. Samples were held at −5 °C for five minutes before controlled nucleation using forceps pre-cooled in liquid nitrogen. This controlled nucleation is performed to ensure that ice nucleation occurs at the same sub-zero temperature of −5 °C in each vial. The samples were held at −5 °C for an additional five minutes before being cooled to −40 °C (1 °C/min). Upon stabilization at −40 °C, the samples were rapidly thawed in a 37 °C water bath and the percentage of intact RBCs was measured. These freezing conditions were repeated two to sixteen times (n = 2–16) for each freezing solution. Error bars are reported as the standard error of the mean (SEM). Asterisks (*) indicate significant difference determined by unpaired Student’s t-test (p < 0.05) compared to 15% glycerol control.

Each of these compounds was investigated for the ability to prevent cryo-injury during freezing and thawing. For these studies, human RBCs were utilized because they are annucleate and assays to reliably assess levels of post-thaw hemolysis are well established. Prior to performing cryomicroscopy with RBCs in the presence of IRIs **3**–**5**, optimal *in vitro* concentrations for each IRI were determined using the two-step rate-controlled freezing experiments^16^. Given that one objective of this study was to reduce the amount of glycerol used during freezing to ultimately reduce post-thaw processing times, a 15% glycerol solution was used instead of 40% (clinical standard). During cryopreservation, RBCs are frozen and stored at −80 °C, at which biochemical reactions do not occur^25^. To achieve successful cryopreservation of RBCs, efforts typically target avoidance of the freezing injury that occurs during slow and fast cooling^26^. For RBCs, the optimal cooling rate is exceptionally high, but can be shifted to slower more practical cooling rates when cryoprotective agents are used. In our initial experiments, RBCs are cooled to −5 °C, the sample is nucleated using a liquid-nitrogen cooled probe which is touched to the outside of the glass vial. This controlled nucleation is performed to ensure that ice nucleation occurs at the same sub-zero temperature of −5 °C in each vial. The sample is cooled at a rate of 1 °C/min to −40 °C and allowed to stabilize at −40 °C. The sample is then warmed rapidly to room temperature and the percentage of intact RBCs is determined. The data in Fig. 4 demonstrates the potential of IRIs **3**–**5** to preserve RBCs using only 15% glycerol during slow cooling. It is interesting to note that the required “dosage” of each IRI is very different (optimal concentrations of **3**–**5** are 110, 30 and 5 mM respectively). As shown in Fig. 4, all three IRIs are very effective at protecting RBCs from cryo-injury and increasing the amount of intact RBCs relative to the 15% glycerol control (p < 0.05 represented by asterisks). It is also interesting to note that **4** is effective at only 30 mM, while the optimal concentration of **3** is 110 mM. This is not surprising as **4** is approximately twice as active as **3** with respect to IRI activity. However, **5** is equally effective (p > 0.05) as **4** at only 5 mM but is less IRI active than both **3** and **4** suggesting that factors other than IRI activity may be important for the cryoprotective activity. The ability to reduce the amount of glycerol in the presence of an IRI without compromising the number of RBCs recovered is significant because cryopreservation using less glycerol will reduce post-thaw processing time in the clinical setting.

**Figure 5:**
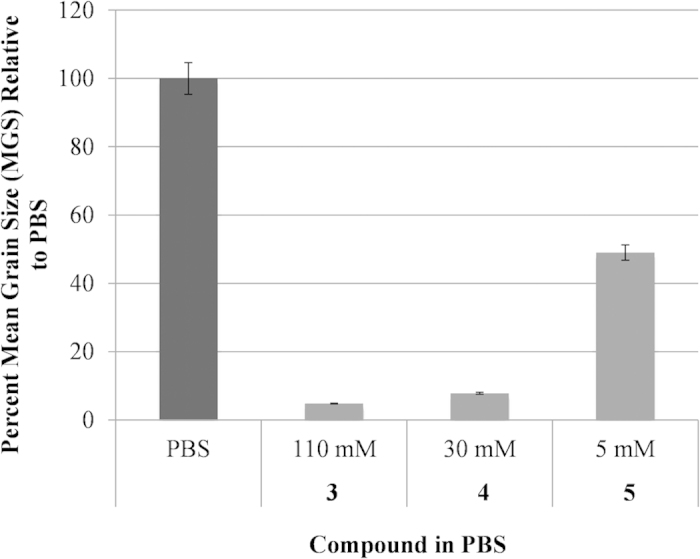
IRI activity of small molecules **3**–**5** at optimized concentrations utilized in the freezing of RBCs. IRI activity is represented as a percent mean grain size (% MGS) after 30 minutes of recrystallization at −6.4 °C compared to a phosphate buffered saline (PBS) positive control for ice recrystallization.

Given that the optimal concentrations for the freezing of RBCs were not the same as those utilized for the assessment of IRI activity (22 mM), the IRI activity of **3**–**5** was re-assessed at their effective *in vitro* concentrations reported in Fig. 4. These data are presented in Fig. 5. As expected, the IRI activity of **4** does not change dramatically, however ice crystal size in the presence of 110 mM **3** is dramatically smaller in size. Interestingly, **5** appeared to be less sensitive to concentration effects as there is little difference in ice crystal size despite the fact the concentration is approximately four-fold less.

### Mean Ice Crystal Size upon Thawing Frozen RBCs

**Figure 6:**
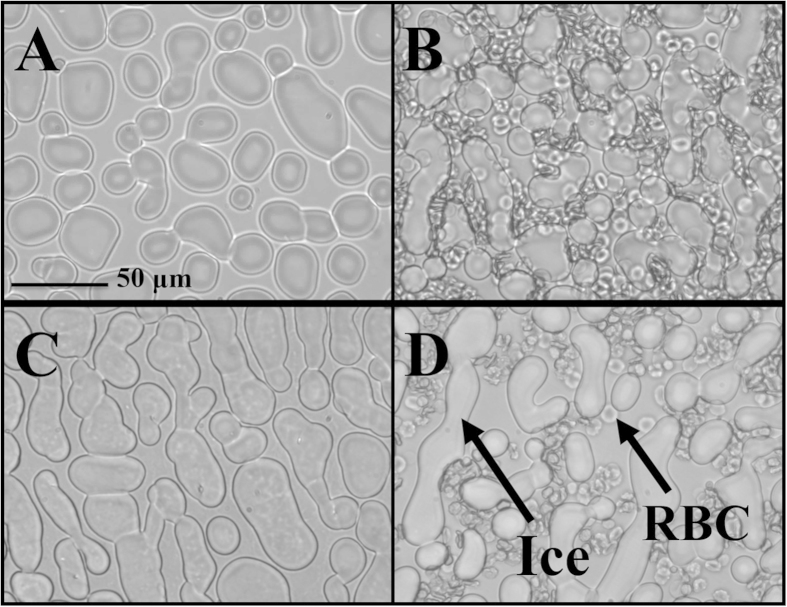
Images of ice in presence of (**A**) 15% glycerol, (**B**) 15% glycerol + RBCs, (**C**) 15% glycerol + 30 mM **4** and (**D**) 15% glycerol +30 mM **4** + RBCs. Samples were cooled to −40 °C (25 °C/min) and then warmed (10 °C/min) to −10 °C. Images shown are after 10 minutes at this temperature.

Analysis of ice crystal size is a key aspect of the splat IRI assay that our laboratory has developed^27^. Previous work from our laboratory has demonstrated that adding small molecule IRIs increases post-thaw viability and allows for a reduction in the amount of cryoprotectants^4^,^10^,^16^,^28^. We predicted that ice crystals should be noticeably smaller in size in the presence of an ice recrystallization inhibitor. Thus, an experiment was performed in which human RBCs were frozen using a Linkam Cryostage and the ice was imaged in the presence of cells. Using this approach, a solution of RBCs in 15% glycerol or 15% glycerol with **4** (30 mM) was cooled at a rate of 25 °C/min to a temperature of −40 °C. The sample was then warmed to −10 °C at a rate of 10 °C/min and held for 10 minutes prior to taking a picture. Figure 6 shows the images of ice crystals in a 15% glycerol solution with and without RBCs. It is important to note that the percentage of ice in the 15% glycerol and 15% glycerol with 30 mM **4** samples stays constant even in the presence of RBCs. In other words, the presence of RBCs does not appear to influence the amount of ice in the sample. However, less ice is observed in samples (with and without RBCs) when **4** (30 mM) is present compared to the 15% glycerol control. In each of these images, the percentage of frozen fraction is small. This is because the holding temperature of −10 °C is close to the colligative freezing point depression of the 15% glycerol solution (−4 °C) and therefore a large fraction of the sample is unfrozen^29^.

**Figure 7:**
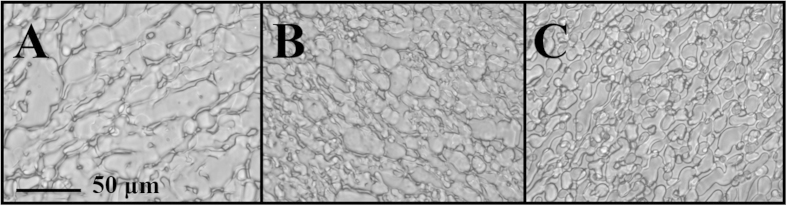
Images of ice in the presence of RBCs with (**A**) 15% glycerol, (**B**) 30 mM **4** in 15% glycerol and (**C**) 5 mM **5 **in 15% glycerol. Samples were cooled to −40 °C (25 °C/min) and then warmed (10 °C/min) to −20 °C. Images shown are after 10 minutes at this temperature. Mean grain size and percentage of ice in the sample is smaller when IRI **4** (30 mM) (**B**) or **5** (5 mM) (**C**) is present in the 15% glycerol solution compared to the 15% glycerol control (**A**).

As the IRI activity of **3**–**5** was assessed in conditions with high amounts of ice present, the experiment was repeated at a lower holding temperature to ensure higher ice volume. Figure 7 shows images of ice crystal size when RBCs were cooled to −40 °C at a rate of 25 °C/min and then warmed at the same rate to −20 °C and held for 10 minutes at this temperature. It is immediately evident that there is a higher ice/water ratio and this more closely mimics the situation in a frozen sample. In the presence of RBCs, the ice crystal size is clearly reduced in samples with IRIs **4** and **5** relative to the 15% glycerol control. Analysis using domain recognition software^27^ indicated that the percent of frozen fraction in each image was 92, 86 and 70% respectively. Thus, there is a progressive increase in the percentage of unfrozen fraction in the presence of IRIs **4** and **5**. This is interesting as it has been hypothesized that cellular injury in slowly frozen red cells is a result of solution effects (solute/electrolyte concentration, severe dehydration) and the reduction of unfrozen fraction in the extracellular space.

### Exacerbating Cellular Injury from Ice Recrystallization

**Figure 8:**
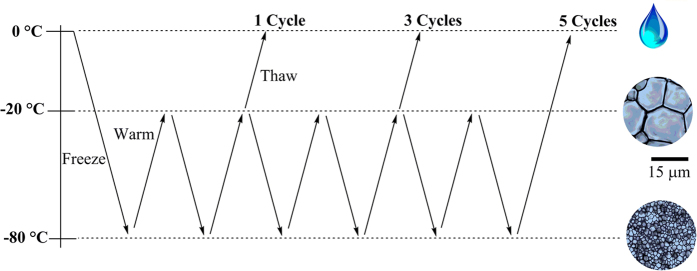
Illustration of how to exacerbate ice recrystallization related injury and transient warming effects. Each −80 °C to −20 °C to −80 °C represents one cycle of transient warming.

Given the fact that the addition of IRIs **3**–**5** resulted in significantly smaller ice crystals *in vitro*, we sought to increase the extent of ice recrystallization in the frozen sample controls. To do this, an experiment was performed in which a sample was frozen by placing it directly in dry ice (dump freeze) at −80 °C (cooling rate of 90 °C/min) and then warmed to −20 °C. After stabilization at −20 °C, the sample was cooled again to −80 °C. This process could be repeated for several cycles (1, 3 or 5 times) before being thawed (see Fig. 8). Dump freeze conditions (fast freezing rate) were chosen in order to maximize the amount of RBCs that would survive using only 15% glycerol and thus it would be easier to observe a reduction in the percentage of intact RBCs cells after several cycles of transient warming.

**Figure 9:**
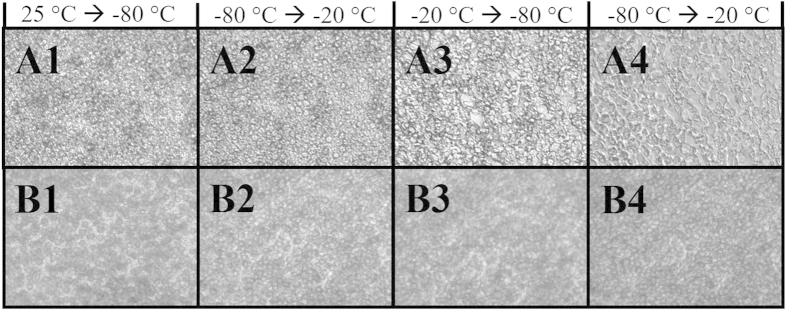
Images of frozen RBCs in 15% glycerol (A1–A4) and RBCs in 15% glycerol with 30 mM **4** (B1–B4). Freezing and warming rates were 90 °C/min and each phase of the cycle (RT to −80 °C, −80 °C to −20 °C etc.) was held for 15 minutes. Images were taken at the end of each phase at the same temperature and time for each sample. In the presence of 30 mM **4**, the ice crystal sizes throughout the experiment remained constant after warming to −20 °C and repeated cycling (panels B1–B4). In the absence of an inhibitor, ice crystal sizes begin to increase after cycling (panels A1–A2 compared to A3–A4).

As the rate of ice recrystallization will increase at higher subzero temperatures, control samples containing only 15% glycerol subjected to repeat warming and cooling cycles were expected to have significantly larger ice crystals prior to thawing than samples with IRIs. To test this concept, a cycling experiment was performed using a cryomicroscope where the sample was cooled to −80 °C rapidly (90 °C/min). The sample was held for 15 minutes and then quickly warmed to −20 °C (90 °C/min) and held for an additional 15 minutes before being cooled to −80 °C again and then warmed to −20 °C prior to thawing. Images were acquired throughout the temperature cycling. The data from this experiment are shown in Fig. 9.

A remarkable aspect of this experiment is that in the presence of 30 mM **4**, the ice crystal sizes throughout the experiment remained constant after warming to −20 °C and repeated cycling (Fig. 9, panels B1–B4). In fact, the average ice crystal size does not change from the initial ice crystal size observed upon initial freezing to −80 °C. On the other hand, in the absence of an inhibitor ice crystal sizes begin to increase after cycling (Fig. 9, panels A1–A2 compared to A3–A4). It is impressive that the IRIs have the ability to control and prevent subsequent ice crystal growth upon warming the frozen sample. The experiment with the 15% glycerol control (Fig. 9, panel A4) is representative of the TWEs when samples are being transferred to and from freezers and between blood banks. TWEs have been recognized as a significant contributor to reduced post-thaw viabilities in sperm^17^, placental cord blood^18^,^19^,^20^, peripheral blood mononuclear cells^21^,^22^, and tissue allographs^23^.

**Figure 10:**
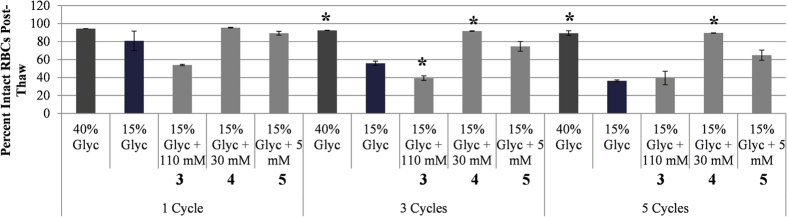
Percentage of intact RBCs after transient warming injury utilizing different cryosolutions – 40% glycerol, 15% glycerol and 15% glycerol with either 3 (110 mM), 4 (30 mM,) or 5 (5 mM). Samples were frozen by placement of vials in dry ice (−80 °C), partially thawed to −20 °C and allowed to stabilize at −20 °C. The samples were then refrozen to −80 °C by again placing the vials in dry ice. This represents one cycle of transient warming (−80 °C to −20 °C to −80 °C). After 1, 3 and 5 cycles of transient warming, the samples were thawed in a 37 °C water bath and percent intact RBCs was measured. These freezing conditions were repeated two to six times (n = 2–6) for each freezing solution. Error bars are reported as the standard error of the mean (SEM). Asterisks (*) indicate significant difference determined by unpaired Student’s t-test (p < 0.05) compared to 15% glycerol control.

Based upon the observations that IRIs can greatly reduce the ice crystal size and that ice recrystallization results in significant decreases in post-thaw cell viability, we hypothesize that inhibiting ice recrystallization during freezing and thawing/warming will result in increased post-thaw cell viabilities. Consequently, we predict that the use of compounds **3**–**5** at the optimized concentrations during freezing of RBCs with only 15% glycerol using slow freezing rates will yield a higher number of intact RBCs post-thaw compared to only 15% glycerol. RBCs were frozen with **3**–**5** at the optimized concentration in 15% glycerol. Samples were cooled rapidly using “dump freeze” conditions (fast freezing rates). After freezing to −80 °C, the sample was then warmed to −20 °C and after stabilization at −20 °C, the sample was cooled again to −80 °C. This constitutes one “cycle”. This cycle was repeated 2 and 4 more times prior to thawing and percent intact RBCs post-thaw was measured corresponding to 1, 3 and 5 freezing-warming-freezing cycles (Fig. 10). Percentage of intact RBCs was compared to a 40% and 15% glycerol control. From the data in Fig. 10, it is apparent that after one freezing-warming-freezing cycle, the 40% and 15% glycerol controls result in very little hemolysis. However, compound **3** is less effective resulting in only approximately 50% intact RBCs. This value is very similar to what is observed in the rate controlled freezing experiments^16^. In contrast, IRIs **4** and **5** are as effective as the 40% glycerol control. When the freezing-warming-freezing cycles are repeated three times (increasing the amount of ice recrystallization in the sample) the percentage of intact RBCs decreases dramatically for the 15% glycerol control (55% intact RBCs) and for compound **3** (40% intact RBCs). The percentage of intact RBCs frozen using 15% glycerol with 30 mM **4** stays constant (92% intact RBCs). When the freezing-warming-freezing cycles are repeated five times, the percent intact RBCs frozen with 15% glycerol and 110 mM **3** is only 36% and 39% respectively. 15% glycerol with 5 mM **5** decreases by 10% to 65% intact RBCs but with IRI **4** (30 mM) the percentage of intact RBCs holds at approximately 90%. With each successive freezing-warming-freezing cycle, the amount of ice recrystallization is increased and the amount of cellular damage is also increased. This is the reason why we observe a decrease in the number of percent intact RBCs with the 15% glycerol control. Interestingly, the 40% glycerol control protects the RBCs against this transient warming injury. Small molecule IRI **4** at 30 mM is a very effective inhibitor of ice recrystallization, in fact it is the most potent inhibitor examined in this study and is also very effective at preventing the cellular injury resulting from ice recrystallization during TWEs with reduced amounts of glycerol.

## Discussion

Ice recrystallization is a major cause of cellular damage during freezing. Conventional cryoprotectants such as DMSO and glycerol function by a number of different mechanisms but do not mitigate or control ice recrystallization. Novel small molecule IRIs control the growth of ice and recrystallization during freezing and unlike the many polymers that are reported to inhibit ice recrystallization, have low molecular masses and are readily amenable to cellular systems. Small molecule IRIs **3**–**5** are effective inhibitors of ice recrystallization in the presence of human RBCs and result in 70–80% intact RBCs post-thaw with reduced amounts of glycerol. The 15% glycerol control furnishes only 40% intact RBCs post-thaw. The ability of **3**–**5** to reduce the mean grain size of extracellular ice was verified by cryomicroscopy and validates the positive correlation between inhibiting the process of ice recrystallization and increased post-thaw recovery of RBCs. Finally, compound **4**, the most effective inhibitor of ice recrystallization in this study was shown to prevent cellular injury due to ice recrystallization during TWEs further demonstrating the utility of these novel small molecule ice recrystallization inhibitors as cryoprotectants.

## Methods

All methods were carried out in accordance with approved guidelines.

### Preparation of aryl-glycosides (3 and 4) and aryl-aldonamide (5)

Aryl-glycosides **3** and **4** were prepared as described previously by our laboratory^16^. *N*-(4-chlorophenyl)-D-gluconamide **5** was synthesized as follows. To a solution of D-gluconic acid-*d*-lactone (0.20 g, 1.12 mmol) in acetic acid (5 mL) was added 4- chloroaniline (0.12 mL, 1.12 mmol). The mixture was stirred under reflux for 2 hours. The crude product was precipitated with hexanes, filtered and the crude solid was recrystallized in EtOH to afford **5** as white crystals (180 mg, 52%); ^1^H NMR (400 MHz, DMSO-*d*_*6*_): δ 9.7 (s, 1H), 7.8 (d, J = 9.1 Hz, 2H), 7.35 (d, J = 8.8 Hz, 2H), 5.71 (d, J = 5.3 Hz, 1H), 4.59 (d, J = 4.9 Hz, 1H), 4.55–4.53 (m, 2H), 4.36 (t, J = 5.7 Hz, 1H), 4.18 (dd, J = 5.1, 3.7 Hz, 1H), 4.02–3.99 (m, 1H), 3.61–3.57 (m, 1H), 3.52–3.51 (m, 2H), 3.42–3.36 (m, 1H); ^13^C NMR (400 MHz, DMSO-*d*_*6*_): δ 171.77, 137.56, 128.40, 126.89, 121.17, 74.20, 72.19, 71.53, 70.31, 63.27; LRMS (ESI) (m/z): [M + Na]^+^ calcd. for C_12_H_16_ClNaNO_6_, 328.70; found, 327.95.

### Ice Recrystallization Inhibition (IRI) Activity

Sample analysis for IRI activity was performed using the “splat cooling” method as previously described^30^. All carbohydrate derivatives assessed were dissolved in a phosphate buffered saline (PBS) solution comprised of sodium chloride (8% w/v), disodium phosphate (1.44% w/v), potassium chloride (0.2% w/v) and monopotassium phosphate (0.24% w/v) in distilled water adjusted to pH 7.4 with concentration hydrochloric acid. A 10 μL droplet of this solution was dropped from a micropipette through a two meter high plastic tube (10 cm in diameter) onto a block of polished aluminum pre-cooled to approximately −80 °C. The droplet froze instantly on the polished aluminum block and was approximately 1 cm in diameter and 20 μm thick. This wafer was then carefully removed from the surface of the block and transferred to a cryostage held at −6.4 °C for annealing. It is important to note that IRI assays have typically used annealing temperatures ranging from −4 °C to −8 °C. The annealing temperature of −6.4 °C was utilized because this is the standard in our laboratory. After a period of 30 minutes at −6.4 °C, the wafer was photographed between crossed polarizing filters using a digital camera (Nikon CoolPix 5000) fitted to the microscope. A total of three drops for each sample were assayed and three images were taken from each wafer with the area of twelve crystals in each image being quantified. Image analysis of the ice wafers was performed using a domain recognition software (DRS) program^10^. This processing employed the Microsoft Windows Graphical User Interface to allow a user to visually demarcate and store the vertices of ice domains in a digital micrograph. The data was then used to calculate the domain areas. All data was plotted and analyzed using Microsoft Excel. The mean grain (or ice crystal) size (MGS) of the sample was compared to the MGS of the control PBS solution for that same day of testing. IRI activity is reported as the percentage of the MGS (% MGS) relative to the PBS control. Therefore, small percentages represent a small MGS (small ice crystals), which is indicative of high IRI activity. Error bars are reported as the standard error of the mean (SEM).

### Blood Collection and Preparation

All RBC units were obtained from NetCAD (Canadian Blood Services’ Network Centre for Applied Development). Whole blood was collected from healthy volunteers using standardized phlebotomy guidelines approved by Canadian Blood Services (CBS). Informed consent was obtained from all donors. All experimental protocols were approved by NetCAD and CBS. Ethics approvals were obtained from Research Ethics Board (REB) at CBS and the University of Alberta. For cryovial experiments, whole blood units were collected and processed by NetCAD (Vancouver, BC). The whole blood was processed using the buffy coat (BC) method to produce leukocyte reduced SAGM RBC units, which has been previously described^31^. For cryomicroscopy experiments, whole blood was collected by standard phlebotomy techniques into EDTA collection tubes, pooled into a 15 mL conical tube and then processed to obtain the RBCs. Processing was achieved by centrifugation (10 min, 4 °C, 2,200 g) followed by removal of the plasma and BC fractions. The remaining RBCs were then washed twice with 0.9% saline/0.2% dextrose (SD) followed by resuspension of the RBCs in SD to a final hematocrit of 0.50 L/L. The prepared RBCs were used on the same day of preparation.

### RBC Freezing Experiments

The freezing solution consists of a 30% glycerol solution prepared from a commercially available glycerol solution (57 Glycerolyte, Baxter) by diluting it with 0.2%/0.9% dextrose/saline (SD). An equal volume of freezing solution was added to 150 μL of RBCs for a final volume of 300 μL. The final concentrations of all freezing solutions were as indicated in the results and discussion. RBC suspensions were transferred to cryotubes and incubated at room temperature for 10 minutes prior to immersion in a methanol bath cooled to −5 °C. A thermocouple was inserted into a RBC/15% glycerol sample (temperature probe) to monitor temperature at 1 second intervals. Once the internal solution from the temperature probe reached −5 °C, ice nucleation was induced by touching the outside of the glass cryotubes with pre-cooled (in liquid nitrogen) forceps. Controlled nucleation is performed to ensure that ice nucleation occurs at the same sub-zero temperature of −5 °C in each vial. RBC samples were then held at −5 °C for 5 minutes. Samples were then cooled at a rate of 1 °C/min to −40 °C, then thawed immediately by plunging in a 37 °C water bath. Post-thaw hematocrits (Hcts) and percent hemolysis was determined for all freezing experiments by comparing the supernatant hemoglobin concentration to total hemoglobin concentration using the cyanmethemoglobin Drabkin’s method^32^. These freezing conditions were repeated two to sixteen times (n = 2–16) for each freezing solution. Percentage of intact RBCs was graphed in addition to error bars reported as the standard error of the mean (SEM). Statistical significance for all data was determined by unpaired Student’s t-test with a 95% confidence level.

### Transient Warming Experiments

The freezing solution consists of a 30% glycerol solution prepared from a commercially available glycerol solution (57 Glycerolyte, Baxter) by diluting it with 0.2%/0.9% dextrose/saline (SD). An equal volume of freezing solution was added to 150 μL of RBCs for a final volume of 300 μL. The final concentrations of all freezing solutions were as indicated in the results and discussion. RBC suspensions were transferred to cryotubes and incubated at room temperature for 10 minutes prior to immersion in dry ice (−80 °C). A temperature probe was used for temperature measurements at 1 second intervals. Once the internal solution reached −80 ± 2 °C, the samples were immersed in a methanol bath cooled to −20 °C. Once the internal solution reached −20 °C, the samples were plunged into dry ice again. RBC samples were held in dry ice until the internal solution from the temperature probe reached −80 ± 2 °C, after which the samples were either thawed (representing one cycle of transient warming) or immersed in a methanol bath cooled to −20 °C. One, three and five cycles of immersion in a −20 °C methanol bath and dry ice were performed. Samples were thawed quickly by plunging in a 37 °C water bath. Post-thaw Hcts and percent hemolysis was determined for all freezing experiments by comparing the supernatant hemoglobin concentration to total hemoglobin concentration using the cyanmethemoglobin Drabkin’s method^32^. These freezing conditions were repeated two to six times (n = 2–6) for each freezing solution. Percentage of intact RBCs was graphed in addition to error bars reported as the standard error of the mean (SEM). Statistical significance for all data was determined by unpaired Student’s t-test with a 95% confidence level.

### Calculation of Percentage of Intact RBCs

Percent post-thaw RBC integrity was calculated using the measured percent hemolysis values according to the following equation: % post-thaw RBC integrity = −100% hemolysis. Data is represented as the mean percentage of post-thaw RBC integrity for each condition. Error bars are reported as the standard error of the mean (SEM). Statistical significance for all data was determined by unpaired Student’s t-test with a 95% confidence level.

### Cryomicroscopy

The nucleation and growth of extracellular ice in solutions containing the IRI compounds were documented using a cryomicroscope that consists of a Nikon 80i fluorescent microscope with a long working distance condenser and objectives, CCD cameras (Hammamatsu ORCA) interfaced to a personal computer and a convection cryomicroscope stage (Linkam FDCS196).

## Additional Information

**How to cite this article**: Briard, J. G. *et al*. Small molecule ice recrystallization inhibitors mitigate red blood cell lysis during freezing, transient warming and thawing. *Sci. Rep*. **6**, 23619; doi: 10.1038/srep23619 (2016).

## Figures and Tables

**Figure 1:**
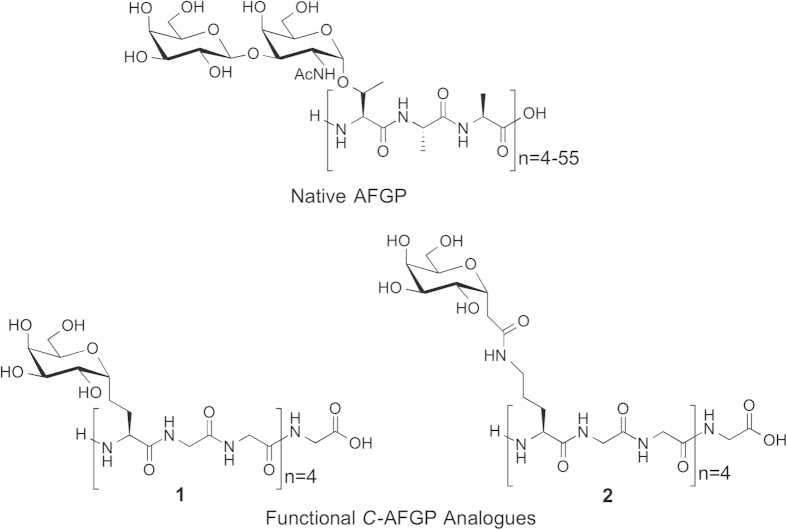
Native antifreeze glycoprotein (AFGP) and *C*-linked AFGP analogues.
